# Mastication stimuli enhance the learning ability of weaning-stage rats, altering the hippocampal neuron transcriptome and micromorphology

**DOI:** 10.3389/fnbeh.2022.1006359

**Published:** 2022-10-03

**Authors:** Akihito Yasuoka, Toshitada Nagai, Seonmi Lee, Hitonari Miyaguchi, Yoshikazu Saito, Keiko Abe, Tomiko Asakura

**Affiliations:** ^1^Department of Applied Biological Chemistry, Graduate School of Agricultural and Life Sciences, The University of Tokyo, Tokyo, Japan; ^2^Department of Human Nutrition, Seitoku University, Chiba, Japan; ^3^Department of Applied Biological Science, Takasaki University of Health and Welfare, Takasaki, Japan; ^4^Kanagawa Institute of Industrial Science and Technology, Kawasaki, Japan

**Keywords:** mastication, powdered diet, hippocampus, memory, dendrite, spine

## Abstract

Mastication stimuli are known to relieve senile dementia in human and animal studies. However, few studies have focused on its effect on weaning-stage animals and the underlying molecular processes. In this study, 3-week-old male rats were raised on a powdered (P-group) or chow (C-group) diet for 8 days, and their behavior was examined using the Y-maze and novel object recognition tests. In the Y-maze test, the C-group rats showed a larger alternation ratio than the P-group rats. In the novel object recognition test, the C-group rats exhibited a significantly larger discrimination index for novel objects than for familiar objects, but the P-group rats did not. We then compared the hippocampal neuron morphology and transcriptome between the groups. C-group rats exhibited larger dendrite branch numbers in the apical dendrites of pyramidal cells in the cornu ammonis 1 (CA1) region and a larger spine density in the basal dendrites of CA1 neurons than the P-group rats. Using DNA microarray analysis, we identified 621 (P < C) and 96 (P > C) genes that were differentially expressed between the groups. These genes were enriched in functional terms related to dendrite growth and included the Igf2, RhoA, and Rho GEF genes, most of which were upregulated in the C-group. These results suggest that the mastication stimuli during the weaning period can enhance the learning ability of rats by increasing the dendrite branches of hippocampal CA1 neurons and by regulating genes related to dendrite growth.

## Introduction

The differential response of animal brain to orosensory stimuli without any change in nutritional supply is an interesting phenomenon. The enhancing effect of mastication stimuli on memory function has been well studied from the perspective of treating senile dementia because the intensity of mastication stimuli tends to reduce with age (Mendes Fde et al., 2013; Chen et al., 2015; Krishnamoorthy et al., 2018). However, it is also important to investigate this effect in the weaning stage because the brain in this stage is more plastic and sensitive to external stimuli than in the adult stage (Alberini and Travaglia, 2017; Matsumoto et al., 2019; Piancino et al., 2020; Rostami et al., 2021). In addition, animals in the weaning stage undergo significant changes in feeding conditions, from breast milk to a solid diet. This process involves changes not only in nutrition, but in the modality of orosensory stimuli as well. During mastication, orosensory stimuli are received at the nerve endings in the oral epithelia, tooth roots, and muscle spindles, and transmitted to the brain to regulate mastication motion. The main ascending pathway goes through the medulla, pons, and thalamus to the somatosensory cortex; however, well-known neuronal connections to other regions such as the hippocampus, amygdala, and frontal cortex, may also be involved in the enhancing effect of memory function (Corson et al., 2012; Fujita et al., 2017; Scheel et al., 2020). In animal models, many researchers have used a powdered diet in contrast to a chow diet as a control to reduce the mastication stimuli, while nutritional equivalence among the experimental groups needs to be carefully maintained. The treatment time window seemed to be a critical part of the experimental design because the window overlaps with the developmental process of brain function after the weaning stage. Several studies have been conducted on weaning-stage rodents, where 3-week-old mice or rats were fed a powdered or liquid diet for more than 7 weeks (Piancino et al., 2020). Patten et al. (2013) raised 3-week-old mice on a liquid or chow diet for 9 weeks and observed an increase in neuronal proliferation in the hypothalamus and hippocampus of the chow diet group. We previously fed 3-week-old male rats a powdered or chow diet for 8 days and analyzed neuronal morphology and transcriptome in the thalamus (Ogawa et al., 2018). Interestingly, the spine numbers of thalamic neurons were significantly decreased in the chow diet group, accompanied by an upregulation of GABA-related gene expression. This was the first study to report that the reduction of mastication stimuli for less than 2 weeks caused changes in neuronal morphology in the thalamus and provided the molecular basis for understanding how the thalamus responds to short-term changes in mastication stimuli strength. These results suggest that mastication stimuli change the proliferation and morphology of the thalamus and hypothalamus, which may influence other brain regions related to memory function, neuroendocrinologically. In this study, we raised male rats at the weaning stage under the conditions described above, and examined the transcriptome and neuronal morphology in the hippocampus with successful detection of significant changes in gene expression and dendrite morphology.

## Materials and methods

### Animals and breeding method

**FIGURE 1:**
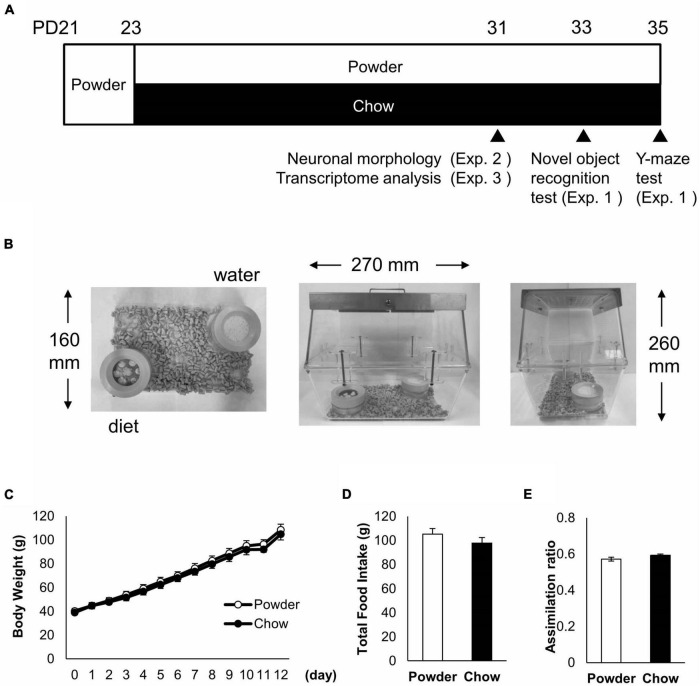
Raising condition and physical parameters of the rats. **(A)** Raising schedule and time points of the behavioral test and tissue sampling. Three-week old (PD21) male rats were acclimatized by feeding a powdered diet and separated into two experimental groups, fed powdered diet (P-group) and chow diet (C-group), respectively. Three independent experiments (Exp. 1 to 3) were conducted for different analyses. **(B)** Raising condition of the rats. Two containers with plastic lids were located symmetrically in the cages made with polycarbonate buckets and metal lids. **(C)** Time course of average body weight in Exp. 1 (*n* = 8 for each group). **(D)** Total food intake from day 2 to 10 in Exp. 1. **(E)** Assimilation ratio from day 2 to 10 in Exp. 1. No significant difference in physical parameters was detected between the experimental groups.

Three-week-old (postnatal day 21, PD21) male Wistar rats (CLEA Japan, Tokyo, Japan) were administered powdered feed and water *ad libitum* for 2 days for acclimatization (Figure 1A). The rats were then divided into two groups so that the two groups of rats weighed the same, and the two groups were fed powdered feed (powder, P) and solid feed (chow, C), respectively. These feeds were the same as those used in a previous study (Ogawa et al., 2018). Rats were housed under controlled conditions at a temperature of 20–26°C and humidity of 40–60% under a 12/12-h light-dark cycle, with *ad libitum* feed and water intake. The light intensity was set to 100 lx. Cages, bedding, and water supplies were designed to avoid chewing, except during diet consumption (Figure 1B). Each cage was made of two polycarbonate standard cages as previously described (Ogawa et al., 2018). The beddings were made of soft paper chips (Eco-chips, CLEA, Japan). Water was administered using a water-absorbent sponge (KS SE10 car wash sponge, Cellulose; Komeri, Niigata, Japan) in a feeding container (Figure 1B).

Three experiments were performed. First, a behavioral test (Exp. 1) was performed. There were 16 rats, eight in the powdered feed group and eight in the solid feed group. They were fed for 10 days after 2 days of conditioned feeding, and then subjected to a novel object recognition test. Two days later, rats were subjected to the Y-maze test. In the second experiment (Exp. 2), neuronal morphology analysis of the hippocampus was conducted in five rats fed for 8 days after conditioned feeding. In the third experiment (Exp. 3), to examine the transcriptome analysis of hippocampus, five rats were fed for 8 days after conditioned feeding. A summary of the feeding schedule is shown in Figure 1A.

This experiment was conducted under strict adherence to the regulations on animal experiments defined by the University of Tokyo, and the study protocol was approved by the ethics board of the university (Approval No. P17-087M03).

### Novel object recognition test

On PD31, the cage containing the rat was moved and placed in the test room for 30 min. The rats were then allowed to explore an open field (50 cm square at the bottom and 50 cm in height; Supplementary Figure 1A) without the objects (Supplementary Figure 1B) for 10 min to allow acclimatization to the test field. This process was repeated on PD32.

On PD33 (the day of the test), the rats were acclimated for 30 min in the test room, and were allowed to explore the field for 10 min. While the rats were moved to a cage in the test room for 5 min, two identical objects (familiar objects; Supplementary Figure 1B) were placed in the field. The rats were allowed to explore the field for 5 min during the acquisition phase. Thirty minutes after staying in the cage, the rat was moved again to the field, where one of the objects was replaced with a different object (novel object, Supplementary Figure 1B), and allowed to explore the field for 5 min as the test phase. The light intensity was set to 35 ± 5 lx throughout the entire process.

The animals’ movements during the acquisition and test phases were recorded for 3 min, 1 min after the objects were placed in the field and were analyzed using SMART 3.0 (Bio Research Center Corporation, Nagoya, Japan). The left and right areas were defined by outlines 3 cm distant from the outer edge of the objects (Supplementary Figure 1A), and the time spent in either area was measured. The discrimination index was calculated using the following formula: (time spent in the area of a novel object – time spent in the area of a familiar object)/time spent in both areas.

### Y-maze test

On PD35, the rats acclimated to the test room were allowed to stay in that room for 30 min and were allowed to explore the Y-maze field for 8 min. Each arm of the maze was 15 × 50 cm at the bottom and 30 cm in height.

Traces of the arm entries were recorded for 5 min after a rat was placed in the maze. One entry was defined as an entry of both hind legs to an arm. The alternation ratio was calculated using the following formula: number of alternations/(total number of arm entries – 2). One alternation was defined as an entry into an arm that was different from the previous two entries.

### Neuronal morphology analysis

To analyze neuronal morphology, Golgi-Cox staining was performed using the SuperGolgi Kit (Bioenno Lifesciences, CA, USA), according to the manufacturer’s protocol. The rats were perfused with 0.9% saline under isoflurane anesthesia (FUJIFILM Wako Pure Chemical Corporation, Osaka, Japan). After perfusion, the brains were removed and blocks containing the hippocampus (1 cm × 2 cm × 1 cm) were immersed in Solution A (Bioenno Lifesciences) and shaken for a day. The solutions were then discarded, and fresh Solution A was added in the same manner. After 9 days, the blocks were washed with distilled water and immersed in Solution B (Bioenno Life Sciences) for a day, and then in B-prepared post-impregnation buffer (Bioenno Life Sciences) for another day.

The blocks were sliced to a thickness of 150 μm using a Vibratome VT1000S system (Leica Biosystems, Wetzlar, Germany). Slices were dried on glass slides, and were subsequently immersed in phosphate-buffered saline with Tween (PBS-T), in Solution C (Bioenno Lifesciences), and in Solution D (Bioenno Lifesciences) for 20 min, respectively. After washing with PBS-T, the air-dried slices were dehydrated with 100% ethanol (Kanto Chemical, Tokyo, Japan) and permeabilized with xylene (Kanto Chemical). Slices enclosed in Entellan New (FUJIFILM Wako Pure Chemical Corporation) were analyzed.

Images of stained sections of the hippocampal cornu ammonis 1 (CA1) region, between bregma −3.80 and −4.52 mm, were captured using an LSM 700 ZEN microscope (Carl Zeiss) while shifting the focal surface by 0.3 μm. Captured images were reconstructed using Neurolucida 11.0 (MBF Bioscience, Williston, VT, USA) by tracing dendrites and spines with lines. Five cell-bodies for each rat (a total of 25 neurons from five rats in one experimental group) were analyzed as to their dendritic morphology. The length, number of branches, and spine density of the entire dendrite derived from a single cell-body were calculated. As there were some branches of dendrites that could not be traced, sample numbers were *n* = 15–25 for each calculation.

### Transcriptome analysis

The hippocampal RNA was extracted and transcriptome analysis was conducted, previously described (Ogawa et al., 2018). Briefly, total RNA was extracted and purified using TRIzol reagent (Thermo Fisher Scientific, Waltham, MA, USA) and an RNeasy Mini Kit (Qiagen, Hilden, Germany). The Affymetrix GeneChip Rat Genome 230 2.0 Array (Thermo Fisher Scientific) was used to obtain the CEL file as raw data. The statistical software R Version 2.7.2 was used to normalize the raw data and perform a hierarchical clustering analysis. Normalization was performed using the factor analysis method for robust microarray summarization, quantile (qFARMS). Two groups were compared using the rank products method using the same software, and probe sets with a false discovery rate (FDR) of < 0.05 were extracted as differentially expressed genes (DEGs). Using the DEGs uploaded to the analytical software, Ingenuity Pathway Analysis (Qiagen), canonical pathways with *p*-value < 0.05 and | Z-score| > 1.5 were extracted as significantly enriched. The CEL files and normalized data were deposited to Gene Expression Omnibus (GSE210292).

### Statistical analysis

The results were shown as the means ± SEMs. One rat in P-group was excluded due to its slow growth rate. No outlier exclusion was applied to the analyses. The paired *t*-test (*p* < 0.05) was used for the novel object recognition test. Other data were analyzed using the Student’s *t*-test (*p* < 0.05).

## Results

### Raising condition of young rats

Three-week old (PD21) male rats were acclimated by feeding a powdered diet for 2 days and maintained under the following experimental conditions: powdered diet (P-group) or chow diet (C-group) for 8 days (Figure 1A). We chose male rats to maintain the consistency with the previous study (Ogawa et al., 2018) and to avoid data perturbation caused by female menstrual cycle in the follow up study that we planned. To reduce spontaneous mastication without diet consumption, the diet containers were covered with plastic lids, and each cage was made of two polycarbonate buckets (Figure 1B). Rats exhibited no difference between the P and C-groups in terms of body weight gain, total food intake, or assimilation quotient (as the ratio of body weight gain to total food intake) (Figures 1C–E). This indicated that the P-condition did not cause a drastic change in the growth of the rats compared with the C-condition.

### Effect of mastication on animal behavior

**FIGURE 2:**
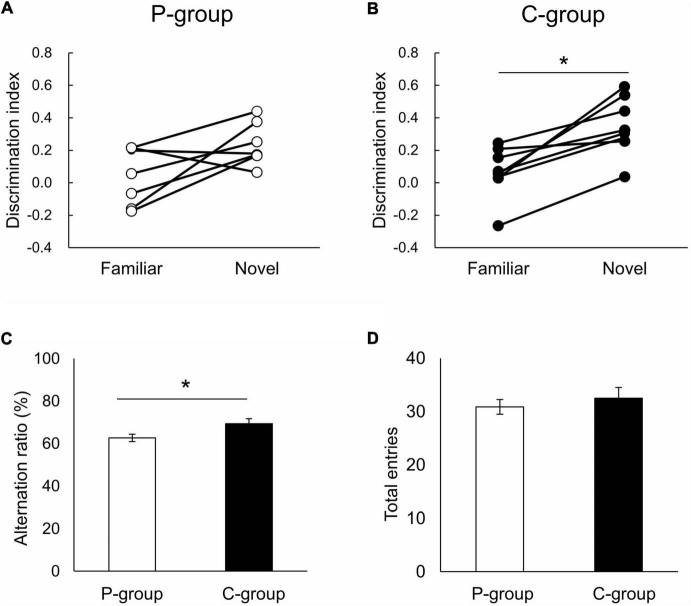
Effect of mastication on learning ability. **(A,B)** Result of the novel object recognition test. Significant difference in discrimination index was detected in the C-group between familiar and novel objects (^⋆^ *p* < 0.05 by paired *t*-test). P-group, *t* (6) = 2.2806, *p* = 0.0627; C-group, *t* (7) = 4.6908, *p* = 0.0022. **(C,D)** Result of the Y-maze test. C-group rats exhibited significantly larger alternation ratio than P-group rats [^⋆^ *p* < 0.05 by *t*-test, *t* (14) = 2.2938, *p* = 0.0378]. No between-group difference was observed in total arm entries [*t* (14) = 0.6706, *p* = 0.5134]. Sample numbers were *n* = 7 for the P-group (one rat excluded because of slow growth), and *n* = 8 for the C-group.

We then examined the learning ability of the animals using a novel object recognition test and Y-maze test. In the novel object recognition test, a novel object was presented to the rats 30 min after learning with two familiar objects (Supplementary Figures 1A,B), and the animals’ ability to memorize objects was evaluated using a discrimination index calculated as the difference in retention time within the area of interest and total retention time near the objects. As a result, C-group rats exhibited a higher discrimination index for a novel object than a familiar object [*t* (7) = 4.6908, *p* = 0.0022 on paired *t*-test], while the P-group rats spent an equal time ratio searching for both of the objects [*t* (6) = 2.2806, *p* = 0.0627] (Figures 2A,B). In the Y-maze test, the C-group rats showed a significantly larger alternation ratio than the P-group rats [*t* (14) = 2.2938, *p* = 0.0378 on Student’s *t*-test], while no between-group difference was observed in total arm entries [*t* (14) = 0.6706, *p* = 0.5134] (Figures 2C,D). These results indicated that the C-group rats were able to store both object memory and working memory more efficiently than the P-group rats.

### Effect of mastication on the morphology of hippocampal neurons

**FIGURE 3:**
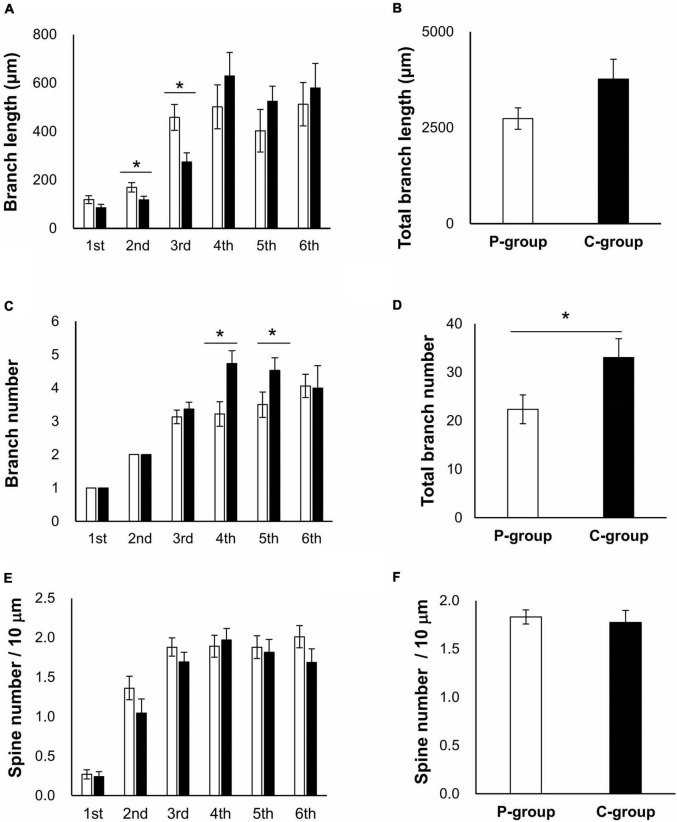
Effect of mastication stimuli on the apical dendrites of CA1 pyramidal cell. **(A)** Branch length of dendrites. Branch numbering is illustrated in Supplementary Figure 2. *: significant differences detected by Student *t*-test [*t* (41) = 2.0526 and *p* = 0.0465 for the second branch, and *t* (41) = 2.8105 and *p* = 0.0075 for the third branch]. **(B)** Total branch length of dendrites. **(C)** Branch number of dendrites. *: significant differences detected by Student *t*-test [*t* (42) = 2.7232 and *p* = 0.0094 for the fourth branch and *t* (39) = 2.3540 and *p* = 0.0237 for the fifth branch]. **(D)** Total branch number of dendrites. *: significant differences detected by Student *t*-test [*t* (42) = 2.2189 and *p* = 0.0319]. **(E)** Spine number per branch length. **(F)** Total branch density per dendrite length. 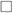: *P*-group, 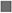: C-group. Sample numbers were *n* = 15–23 for each calculation.

**FIGURE 4:**
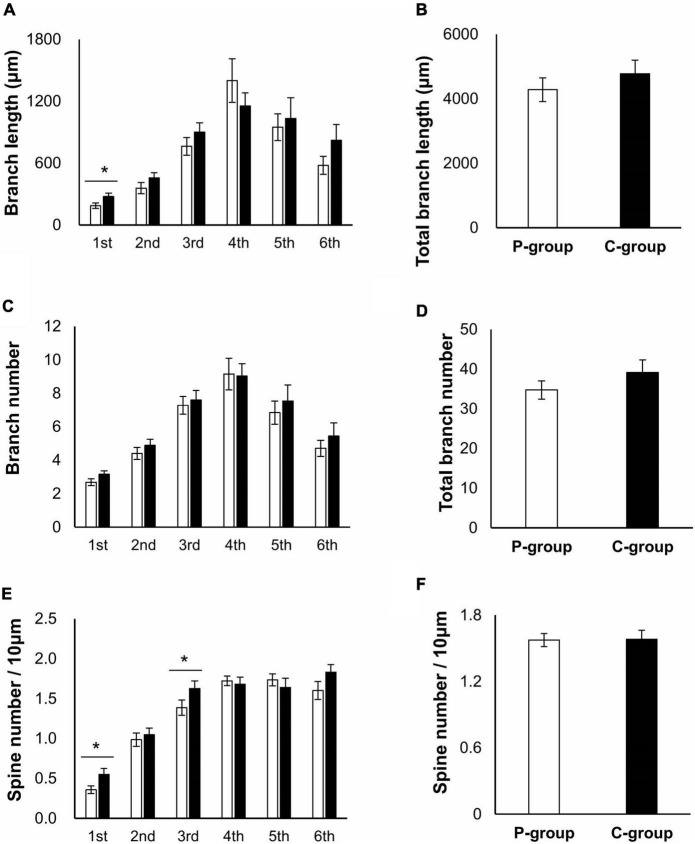
Effect of mastication stimuli on the basal dendrites of CA1 pyramidal cell. **(A)** Branch length of dendrites. Branch numbering is illustrated in Supplementary Figure 2. *: significant differences detected by Student *t*-test [*t* (48) = 2.2613 and *p* = 0.0283 for the first branch]. **(B)** Total branch length of dendrites. **(C)** Branch number of dendrites. **(D)** Total branch number of dendrites. **(E)** Spine number per branch length. *: significant differences detected by Student *t*-test [*t* (48) = 2.6018 and *p* = 0.0123 for the first branch and *t* (48) = 2.4425 and *p* = 0.0183 for the third branch]. **(F)** Total branch density per dendrite length. 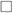: P-group, 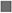: C-group. Sample numbers were *n* = 17–25 for each calculation.

We then speculated that these behavioral phenotypes would correlate with some physiological changes in a brain region involved in memory function, the hippocampus. Brain sections were prepared from the rats raised in the same way as for the behavioral tests (Figure 1A) and subjected to Golgi-Cox staining to evaluate dendrite morphology. We focused on CA1 region of the hippocampus as it is involved in spatial memory processing (Tsien et al., 1996). The apical and basal dendrites of CA1 pyramidal cells were analyzed in terms of both branch number per dendrite and spine number per branch, using a deconvolution software (Supplementary Figure 2). The basal dendrites could be traced up to the sixth branches within the stratum oriens layer, and the apical dendrites could be traced to the sixth branches within the stratum radiatum layer. In the apical dendrites, the length of the second and third branches were significantly longer in the P-group [the second branch, *t* (41) = 2.0526, *p* = 0.0465; the third branch, *t* (41) = 2.8105, *p* = 0.0075 on Student’s *t*-test], while the numbers of the fourth and fifth branches were significantly larger in the C-group [the fourth branch, *t* (42) = 2.7232, *p* = 0.0094; the fifth branch, *t* (39) = 2.3540, *p* = 0.0237] (Figures 3A–D). However, there was no difference in the spine number per 10 μm branch length (spine density) (Figures 3E,F). In the basal dendrites, the length of the first branch was significantly longer in the C-group [*t* (48) = 2.2613, *p* = 0.0283] (Figure 4A). In addition, the C-group exhibited a significantly larger spine density in the first and in the third branches [the first branch, *t* (48) = 2.6018, *p* = 0.0123; the third branch, *t* (48) = 2.4425, *p* = 0.0183] (Figure 4E).

### Regulation of hippocampal transcriptome by mastication

**FIGURE 5:**
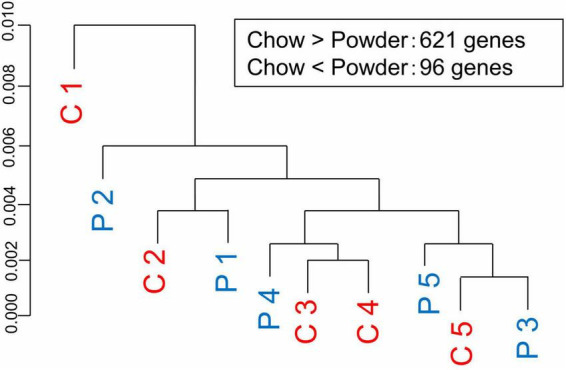
Transcriptome analysis of the hippocampus. Hippocampal regions were manually dissected and subjected to transcriptome analysis using Rat Genome 230 2.0 Array chip. C-group (C 1 to 5) and P-group (P 1 to 5) did not show clear segregation in terms of overall gene expression (cluster dendrogram). Numbers of differentially expressed genes were indicated as an inset.

We then investigated the molecular mechanisms underlying the differences in dendrite morphology between the groups. DNA microarray analysis of hippocampal tissue detected 621 genes upregulated and 96 genes downregulated in the C-group (GSE210292), whereas no overall separation of transcriptomes was observed in the cluster analysis (Figure 5). Ingenuity Pathway Analysis revealed that some DEGs were significantly enriched in terms related to cellular signaling, such as calcium signaling, phospholipase C signaling, and ERK5 signaling (Table 1). We also surveyed all DEGs according to their roles in dendrite growth. As a result, 12 genes appeared to be involved in the cellular signaling related to dendrite growth (Table 2). The overall regulation of these genes was predicted to have a positive effect on dendrite growth in the C-group (9 genes out of 12, Table 2). In particular, Igf2 and Epha4 were identified as the outer cellular signals that can regulate the inner cellular components, such as guanine nucleotide exchange factors (Gef3 and 9) and small G-proteins (Rala, RhoA, and Rnd2). Taken together, these DEGs in hippocampal neurons could be the cause of the differences in dendrite morphology.

**TABLE 1:** Enrichment analysis of differentially expressed genes.

Canonical pathways	−log(*p*-value)	z-score	Regulation	Enriched genes
Calcium signaling	4.45	1.9	P < C	Grin1, Grin2a, Atp2b1, Ryr2, Crebbp, Mef2a, Atp2a2, Atp2b2, Mef2c, Chrna5, Atp2b4, Gria3
			P > C	Chrna5, Ppp3ca
Phospholipase C signaling	2.20	1.51	P < C	Rnd2, Fyn, Rala, RhoA, Pla2g5, Crebbp, Mef2a, Mef2c, Arhgef9
			P > C	Gnb3, Arhgef3, Ppp3ca
Cholecystokinin/gastrin-mediated signaling	1.65	2.45	P < C	Rnd2, RhoA, Mef2a, Mapk8, Epha4, Mef2c
Cardiac hypertrophy signaling	1.49	1.67	P < C	Rnd2, Cacna1d, Kl, RhoA, Crebbp, Mef2a, Mapk8, Mef2c
			P > C	Gnb3, Ppp3ca
Prolactin signaling	1.47	2.00	P < C	Fyn, Kl, Crebbp, Prlr
			P > C	Socs7
ERK5 signaling	1.32	2.00	P < C	Ywhab, Crebbp, Mef2a, Mef2c

Significant enrichment of the terms belonging to canonical pathways of the Ingenuity Pathway Analysis (*p* < 0.05, | Z-score| > 1.5) are listed.

**TABLE 2:** Genes associated with dendrite growth.

Gene name	Dendrite growth regulation	References	FDR	Gene regulation	Predicted effect on dendrite
Insulin-like growth factor (IGF) 2	Up	Schmeisser et al., 2012	1.73E-02	C > P	C-group up
Rho guanine nucleotide exchange factor (GEF) 3 (Arhgef3)	Down	Liao et al., 2021	3.92E-02	C < P	C-group up
Ccd42 guanine nucleotide exchange factor (GEF) 9 (Arhgef9)	Up	de Groot et al., 2017	3.24E-02	C > P	C-group up
Eph receptor A4 (Epha4)	Up	Galimberti et al., 2010; Willi et al., 2012	7.94E-03	C > P	C-group up
FYN proto-oncogene, Src family tyrosine kinase (Fyn)	Up	Babus et al., 2011	4.59E-02	C > P	C-group up
Klotho (Kl)	Up	Vo et al., 2019	2.87E-03	C > P	C-group up
Mitogen-activated protein kinase 8 (Mapk8)	Up	Komulainen et al., 2020	2.89E-02	C > P	C-group up
Myocyte enhancer factor 2a (Mef2a)	Down	Akhtar et al., 2012	3.03E-02	C > P	C-group down
Myocyte enhancer factor 2c (Mef2c)	Down	Akhtar et al., 2012	1.40E-02	C > P	C-group down
v-Ral simian leukemia viral oncogene homolog A, ras related (Rala)	Up	Nakamura et al., 2013	4.56E-02	C > P	C-group up
Ras homolog family member A (RhoA)	Down	Liao et al., 2021	3.12E-02	C > P	C-group down
Rho family GTPase 2 (Rnd2)	Up	Gladwyn-Ng et al., 2016	3.58E-02	C > P	C-group up

## Discussion

It has been shown that hippocampus-dependent memory function matures at the weaning stage in rat. Before weaning, pups are unable to store contextual memory, and adult-like directional and spatial learning begins to appear more than 26 days after birth (Tan et al., 2017; Travaglia et al., 2018). In our study, rats were fed an experimental diet from 23 to 31 days after birth, exactly overlapping the adult-like memory development process. Pups store this kind of memory depending on the sensory stimuli caused by their interaction with the environment, and the reduction of these stimuli, for example, by social isolation, has been shown to impair memory function (Võikar et al., 2005). The question arises as to why mastication stimuli, which is not directly involved in body movement or recognition of circumstances, can enhance memory function. Based on the hypothesis that there can be a neuronal interaction between mastication stimuli and memory processing within the brain, we previously examined the morphology and transcriptome of thalamic neurons because the thalamus is the intersection of sensory stimuli (Ogawa et al., 2018). We found that spine density was decreased, and GABA signals were upregulated in the chow-diet group. In particular, GABAergic signaling seemed to be responsible for memory enhancement by mastication stimuli. The nucleus reuniens (nRE) in the thalamus has been shown to relay cognitive information processing in the prefrontal cortex to the hippocampus. Studies using GABA agonists have revealed that nRE activity is necessary for fear memory extinction and spatial memory retrieval (Dolleman-van der Weel et al., 2019; Lin et al., 2020). Although they used GABA agonists to suppress nRE neurons, these observations are similar to those of infantile amnesia, where infant memories are extinct before the weaning stage (Alberini and Travaglia, 2017). Our results suggest that intrinsic GABAergic signaling in the nRE may play some role in the enhancement of hippocampal memory function by mastication stimuli.

Our transcriptome analysis of the hippocampus suggested the activation of calcium signals and upregulation of signaling components related to dendrite genesis (Tables 1, 2), while the result of morphological analysis seemed to be partly contradictory; a decrease of dendrite length in the apical region was observed in relation to an increase of the branch length and spine number in the basal region (Figures 3, 4). The apical dendrites of CA1 have been shown to receive inhibitory input from nRE, whereas basal dendrites are synapsed on mainly by neurons within the hippocampus (Dolleman-van der Weel et al., 2019). This difference in the innervation properties can cause differences in morphological responses to mastication stimuli between the apical and basal dendrites. Nevertheless, the upregulation of molecules related to growth factor receptor signaling (Tables 1, 2) and the increase in spine number in the basal region (Figure 4) strongly suggest the participation of some kind of a growth factor in the regulation of CA1 dendrite formation. One plausible candidate is brain-derived growth factor (Bdnf), given that there are several reports on the upregulation of Bdnf in the hippocampus and other brain regions upon mastication stimuli using multiple animal models (Chen et al., 2015; Fukushima-Nakayama et al., 2017; Sunariani et al., 2019). In our study, no significant change in Bdnf mRNA expression was observed in the hippocampus or in the thalamus (Ogawa et al., 2018). More importantly, we identified the genes, Igf2 and Epha4, both of which were upregulated in the C-group (Table 2). The expression of Igf2 has been shown to repair stress-induced memory impairments in young rats (Fukushima-Nakayama et al., 2017). Epha4 is responsible for the maintenance of synaptic transmission efficiency and memory function in rodents (Galimberti et al., 2010; Willi et al., 2012). It may be important to examine the protein levels of these outer cellular factors in brain regions and sera using our weaning-stage model.

Taken together, our results provide a molecular clue toward elucidating the enhancement of memory function by mastication stimuli, and highlight the significance of adequate mastication at the weaning stage for brain function. Further neurological studies, such as examining whether GABA-positive neurons in the nRE are responsible for the enhancement of memory function, may shed light on the underlying mechanisms.

## Data availability statement

The data of DNA microarray analysis were deposited to Gene Expression Omnibus (GSE210292). Other datasets generated and analyzed during the current study are available from the corresponding author upon request.

## Ethics statement

The animal study was reviewed and approved by the Ethics Board of The University of Tokyo (Approval No. P17-087M03).

## Author contributions

TA and AY conceived the idea of this study. TN and SL conducted the behavioral experiments and interpreted the data. YS, TN, and HM conducted the neuronal morphological analysis. AY, SL, HM, and YS performed the DNA microarray analysis and interpreted the data. AY, TN, SL, KA, and TA wrote the manuscript. KA and TA supervised the study. All authors contributed to the article and approved the submitted version.

## Abbreviations

Bdnf: brain-derived neurotrophic factor
CA1: cornu ammonis 1
DEGs: differentially expressed genes
Epha4: erythropoietin-producing hepatocellular carcinoma receptor A4
FDR: false discovery rate
GEF: guanine nucleotide exchange factor
Igf2: insulin-like growth factor 2
nRE: nucleus reuniens
PBS-T: phosphate-buffered saline containing Tween-20
PD: postnatal day
qFARMS: the factor analysis method for robust microarray summarization quantile
Rala: rat sarcoma virus (ras) like proto-oncogene A
RhoA: Ras homolog family member A
Rnd2: Rho family GTPase 2.
