# Supplementary material for: Mastication stimuli enhance the learning ability of weaning-stage rats, altering the hippocampal neuron transcriptome and micromorphology
Source: Front Behav Neurosci. 2022 Oct 3;16:1006359. doi: 10.3389/fnbeh.2022.1006359 (PMC9574334; doi:10.3389/fnbeh.2022.1006359)
Supplement: Supplementary file 1 [file Data_Sheet_1.PDF]

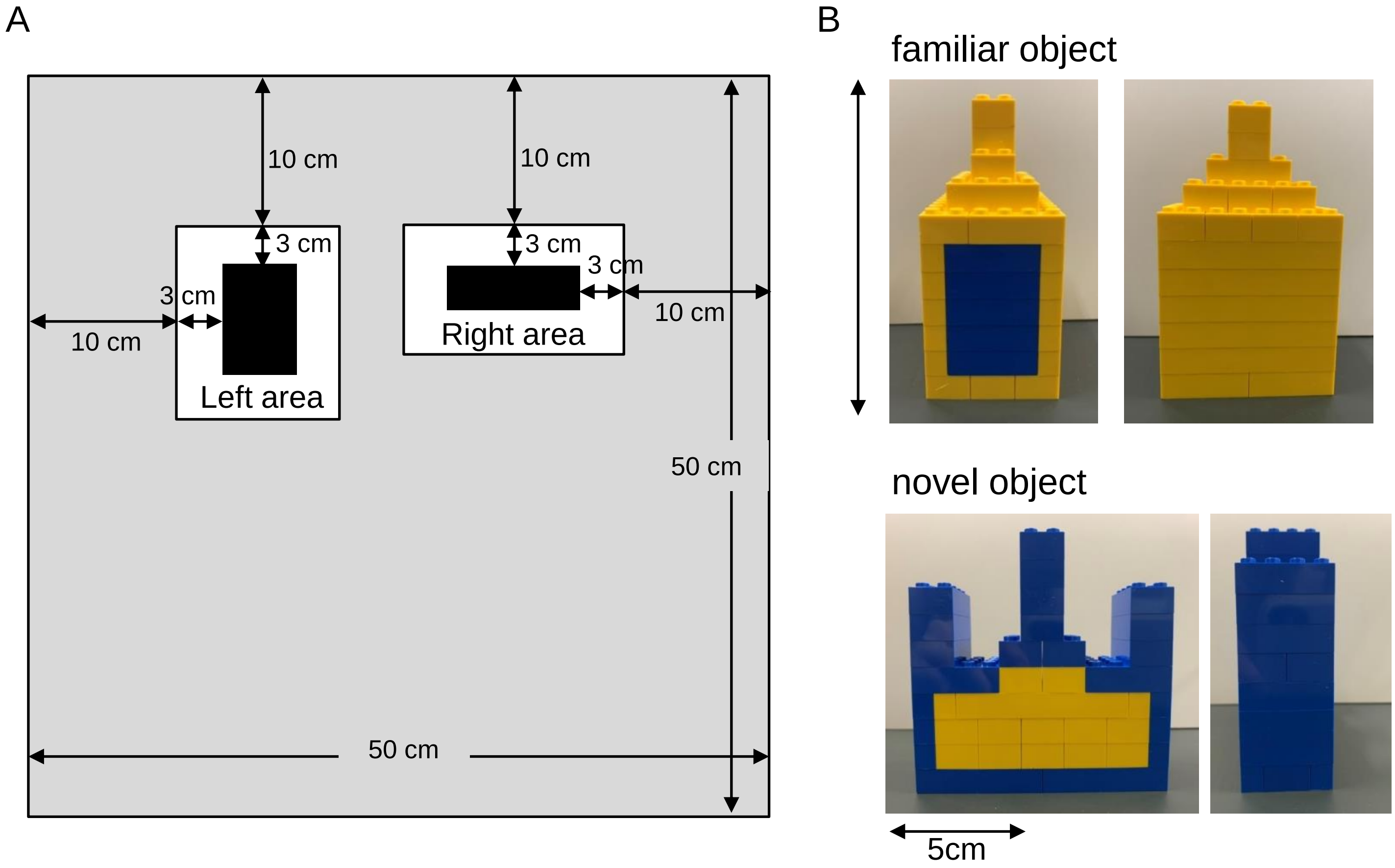

Supplementary Fig. 1. Apparatus for the novel object recognition test **(A)** Field for the test. The objects were located 13 cm distant from the walls. Areas proximal to the objects were defined as a rectangle, 3 cm along and across from the objects. During the learning period, familiar objects were placed in both ‘Left’ and ‘Right’ areas. In the test period, a novel object was located in either of the areas. **(B)** Design of the objects. The objects were similar in color composition, both of which can be recognized by the visual system of rats, but different in shape (smaller than the animal’s height, 12 cm) and color arrangement.

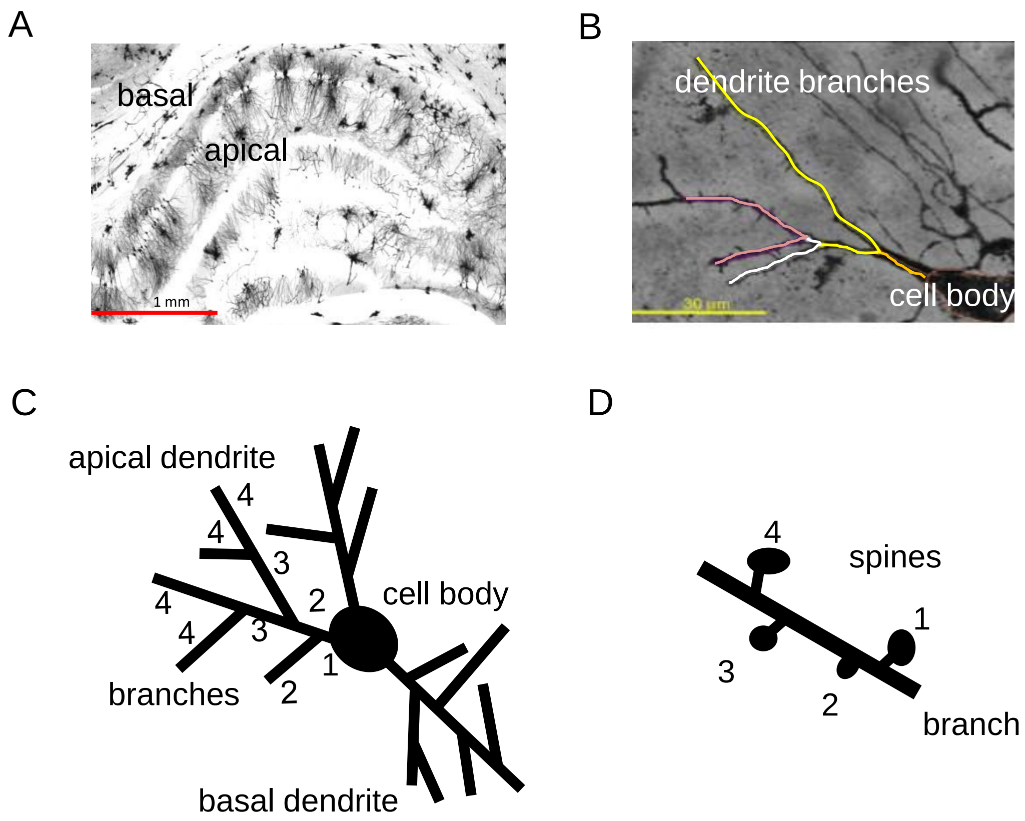

Supplementary Fig. 2. Morphological analysis of hippocampal pyramidal cell

(A) Golgi-Cox staining of the hippocampus at Bregma -4.52 mm coronal section. **(B)** The first (orange), second (yellow), third (white), and fourth (pink) branches are marked in a magnified image of one dendrite. Five pyramidal cells in CA1 per rat were randomly chosen and analyzed. **(C)** Counting scheme of apical dendrites. **(D)** Examples of spines counted.
